# Supplementary material for: A network dynamical simulation model for the study of antibiotic resistance in microbial communities
Source: Gut Microbes. 2026 Sep 19;18(1):2734703. doi: 10.1080/19490976.2026.2734703 (PMC13596919; doi:10.1080/19490976.2026.2734703)
Supplement: Supplementary file 1 [file KGMI_A_2734703_SM9053.zip › supplementary_file_1/Figures/ba_delta_n_components_n1000.pdf]

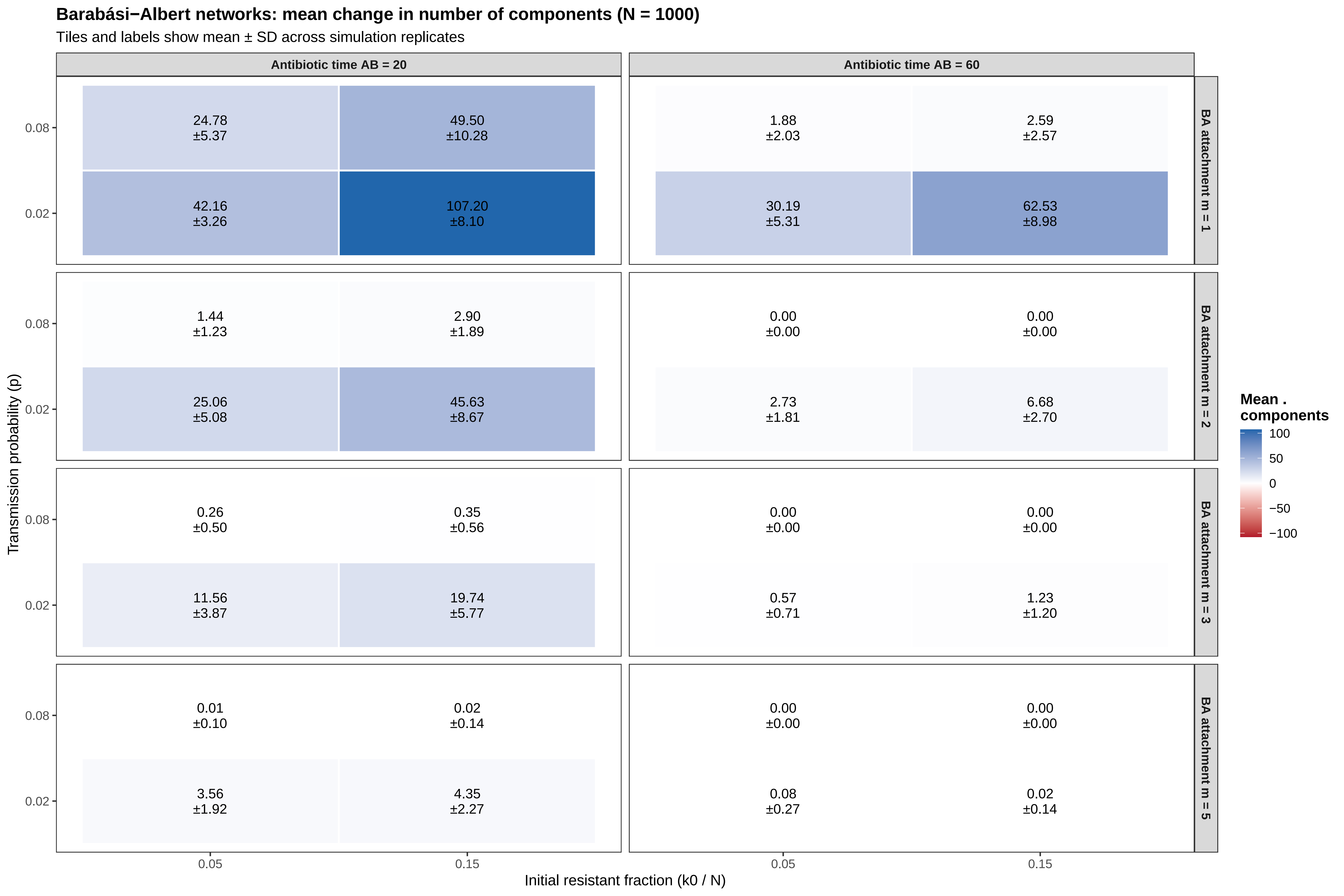

Barabási−Albert networks: mean change in number of components (N = 1000)
Tiles and labels show mean ± SD across simulation replicates
Antibiotic time AB = 20
Antibiotic time AB = 60
BA attachment m = 1
BA attachment m = 2
BA attachment m = 3
BA attachment m = 5
24.78 ±5.37
49.50 ±10.28
42.16 ±3.26
107.20 ±8.10
1.88 ±2.03
2.59 ±2.57
30.19 ±5.31
62.53 ±8.98
1.44 ±1.23
2.90 ±1.89
25.06 ±5.08
45.63 ±8.67
0.00 ±0.00
0.00 ±0.00
2.73 ±1.81
6.68 ±2.70
0.26 ±0.50
0.35 ±0.56
11.56 ±3.87
19.74 ±5.77
0.00 ±0.00
0.00 ±0.00
0.57 ±0.71
1.23 ±1.20
0.01 ±0.10
0.02 ±0.14
3.56 ±1.92
4.35 ±2.27
0.00 ±0.00
0.00 ±0.00
0.08 ±0.27
0.02 ±0.14
0.08
0.02
0.05
0.15
Transmission probability (p)
Initial resistant fraction (k0 / N)
Mean . components
100
50
0
−50
−100
